# The many faces of SIRT6 in the retina and retinal pigment epithelium

**DOI:** 10.3389/fcell.2023.1244765

**Published:** 2023-11-01

**Authors:** Jie Cheng, Casey J. Keuthan, Noriko Esumi

**Affiliations:** Wilmer Eye Institute, Johns Hopkins University School of Medicine, Baltimore, MD, United States

**Keywords:** SIRT6, protein deacylase, retina, retinal pigment epithelium, glucose metabolism, oxidative stress

## Abstract

Sirtuin 6 (SIRT6) is a member of the mammalian sirtuin family of NAD^+^-dependent protein deacylases, homologues of the yeast silent information regulator 2 (Sir2). SIRT6 has remarkably diverse functions and plays a key role in a variety of biological processes for maintaining cellular and organismal homeostasis. In this review, our primary aim is to summarize recent progress in understanding SIRT6’s functions in the retina and retinal pigment epithelium (RPE), with the hope of further drawing interests in SIRT6 to increase efforts in exploring the therapeutic potential of this unique protein in the vision field. Before describing SIRT6’s role in the eye, we first discuss SIRT6’s general functions in a wide range of biological contexts. SIRT6 plays an important role in gene silencing, metabolism, DNA repair, antioxidant defense, inflammation, aging and longevity, early development, and stress response. In addition, recent studies have revealed SIRT6’s role in macrophage polarization and mitochondrial homeostasis. Despite being initially understudied in the context of the eye, recent efforts have begun to elucidate the critical functions of SIRT6 in the retina and RPE. In the retina, SIRT6 is essential for adult retinal function, regulates energy metabolism by suppressing glycolysis that affects photoreceptor cell survival, protects retinal ganglion cells from oxidative stress, and plays a role in Müller cells during early neurodegenerative events in diabetic retinopathy. In the RPE, SIRT6 activates autophagy in culture and protects against oxidative stress in mice. Taken together, this review demonstrates that better understanding of SIRT6’s functions and their mechanisms, both in and out of the context of the eye, holds great promise for the development of SIRT6-targeted strategies for prevention and treatment of blinding eye diseases.

## 1 Introduction

Sirtuin 6 (SIRT6) is a member of the mammalian sirtuin family of nicotinamide adenine dinucleotide (NAD^+^)-dependent protein deacylases, which consists of seven members (SIRT1–7) of homologues of the yeast silent information regulator 2 (Sir2) (Haigis and Guarente, 2006; Chang et al., 2020). SIRT6 has attracted investigators from a wide range of biological fields because of its remarkably diverse functions, such as gene silencing, DNA repair, metabolism, antioxidant defense, inflammation, and longevity (Chang et al., 2020). In addition, more recent studies have revealed further details of SIRT6’s role in macrophage polarization (Zou et al., 2021; Chen J. et al., 2022) and mitochondrial homeostasis (Smirnov et al., 2023). Remarkably, research related to SIRT6 has dramatically increased in the last decade, with the number of publications per year exceeding 100 since 2016 and over 150 since 2020 (PubMed). While the majority of these studies focused on the role of SIRT6 in tissues outside of the eye, important and interesting functions of SIRT6 in the retina and retinal pigment epithelium (RPE) have emerged in recent years.

In vertebrate eyes, the neural retina (simply called the retina) initiates visual signals in photoreceptor cells by converting photons of light to electrical signals (i.e., phototransduction), which are then propagated to other retinal neurons and eventually transferred out of the eye and into the brain (Luo et al., 2008). The function and structure of the retina have been reviewed in great detail (Yau and Hardie, 2009; Hoon et al., 2014). The RPE is a monolayer of polarized epithelial cells residing between the retina and the highly vascularized choroid that supports the survival and functions of retinal photoreceptors in multiple ways, including a blood–retina barrier, the recycling of retinoid chromophore, phagocytosis of photoreceptor outer segments, transfer of nutrients and oxygen, and symbiotic energy metabolism (Strauss, 2005; Lakkaraju et al., 2020; Hurley, 2021). Due to a lack of a direct blood supply to the outer retina, photoreceptor cells utilize glucose as an energy source from the choroidal circulation through the RPE and convert it to lactate, which is then exported to RPE cells as their energy source (Kanow et al., 2017; Hurley, 2021). This symbiotic relationship between the retina and RPE in energy metabolism requires a delicate balancing act of SIRT6 because SIRT6 regulates glucose homeostasis by suppressing glycolysis as described later.

In this Review, we first describe the general functions of SIRT6, except those in cancer, and then summarize the recent progress in understanding the specific functions of SIRT6 in the retina and RPE. For the details of SIRT6’s general functions and roles in physiology and diseases, please refer to a comprehensive review published recently (Chang et al., 2020).

## 2 Multiple functions of SIRT6

**FIGURE 1:**
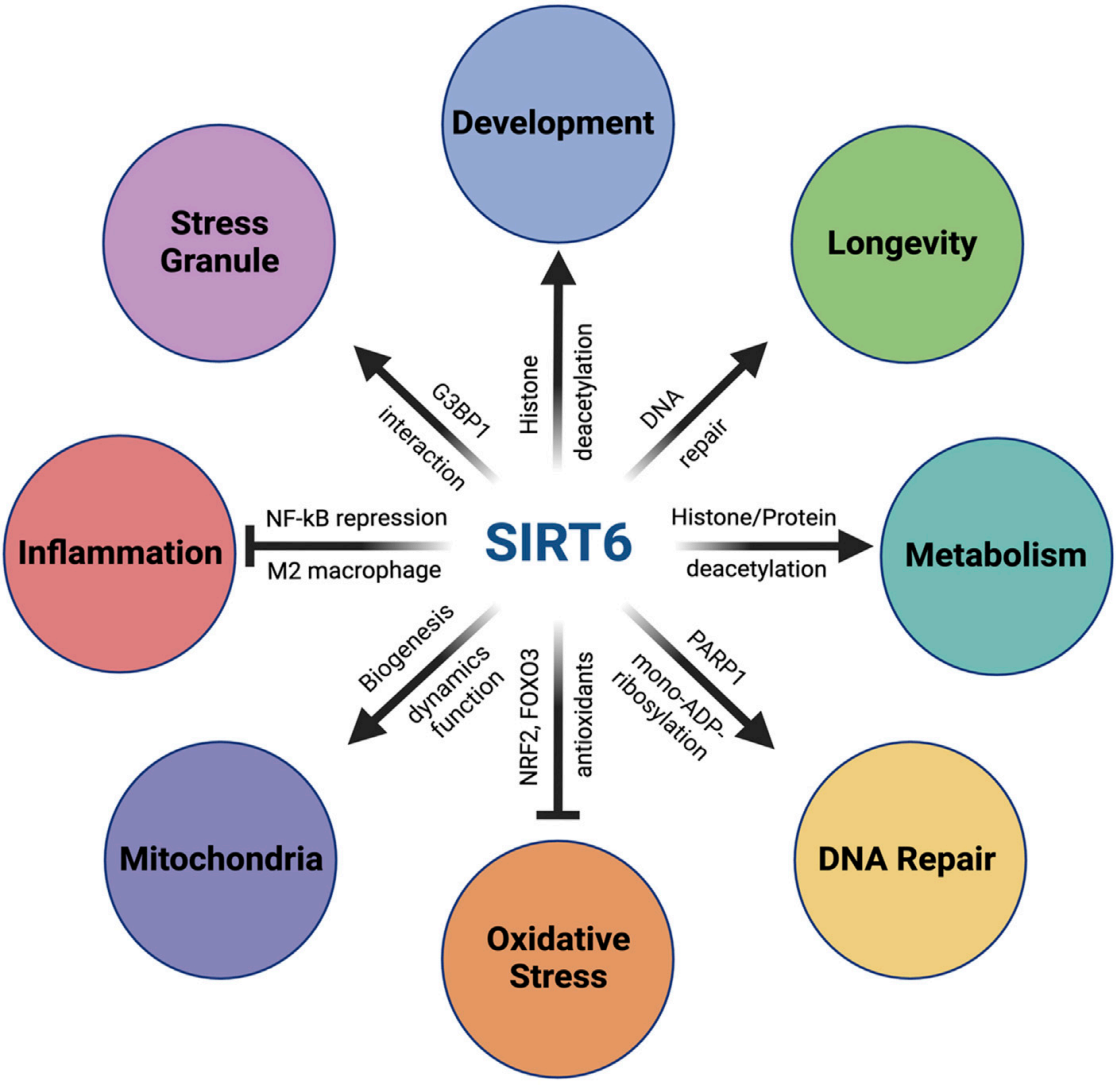
Multiple functions of SIRT6. SIRT6 has multiple functions and plays a key role in a variety of biological processes for maintaining cellular and organismal homeostasis. In metabolism, SIRT6 plays a critical role in glucose homeostasis by repressing glycolysis-related genes through its function as a histone deacetylase on histone H3K9 at these genes. SIRT6 also modulates lipid metabolism through histone/protein deacetylation. In DNA repair, SIRT6 enhances both DNA double-strand breaks (DSB) repair and base excision repair (BER) through activation of PARP1 by mono-ADP-ribosylation. In addition, SIRT6 also facilitates nucleotide excision repair (NER). Importantly, SIRT6’s activity to promote DSB repair is correlated with the lifespan of rodents, suggesting the importance of the SIRT6–DNA repair axis in longevity. In response to oxidative stress, SIRT6 upregulates antioxidant enzymes by acting as a coactivator of NRF2, a key transcriptional regulator of antioxidant defense, as well as by activating FOXO3 in an NRF2-independent mechanism. SIRT6 also plays a positive role in mitochondrial biology, including biogenesis, fusion–fission dynamics, and function. With inflammatory stimuli, SIRT6 interacts with p65 of NF-κB, a master regulator of inflammation, and suppresses expression of NF-κB target genes through H3K9 deacetylation. SIRT6 also exerts anti-inflammatory function by promoting macrophage polarization toward the M2 phenotype. Additionally, SIRT6 also plays a critical role during early embryonic development. SIRT6-null monkeys show severe prenatal developmental retardation and die just after birth, with elevated *H19* expression and higher levels of acetylated H3K56. Human fetuses carrying SIRT6 mutations lacking deacetylase activity show severe neurodevelopmental and cardiac anomalies and die before birth. In addition, SIRT6 also regulates the assembly and disassembly of stress granules (SGs) in the cytoplasm. In response to various stresses, SIRT6 quickly translocates from the nucleus to cytoplasm and interacts with G3BP1, a key component of SGs. Furthermore, SIRT6 has been found to play complicated roles in cancer as both an oncogene and a tumor suppressor, which is not included in this review.

SIRT6 has multiple functions and plays a key role in a variety of biological processes for maintaining cellular and organismal homeostasis, which are described below and summarized in Figure 1.

### 2.1 Enzymatic activities of the SIRT6 protein

SIRT6 is a multi-functional nuclear protein that acts as an NAD^+^-dependent protein deacetylase, deacylase, and mono-ADP ribosyltransferase in a variety of biological processes, including inflammation, metabolism, oxidative stress, DNA repair, genomic stability, aging, and longevity (Mostoslavsky et al., 2006; Chang et al., 2020). In some of these processes, SIRT6 functions through gene silencing and chromatin regulation as a Class III histone deacetylase (HDAC) that targets acetylated histones, particularly acetylated histone H3 lysine 9 (H3K9Ac), lysine 56 (H3K56Ac), and lysine 18 (H3K18Ac) (Michishita et al., 2008; Kawahara et al., 2009; Michishita et al., 2009; Kawahara et al., 2011; Tasselli et al., 2016). In addition, SIRT6 interacts with key biological regulators such as nuclear factor kappa B (NF-κB), hypoxia-inducible factor-1 subunit alpha (HIF1α), poly(ADP)-ribose polymerase 1 (PARP1), and nuclear factor erythroid 2 like 2 (NFE2L2, also known as NRF2) to modulate these pathways and their downstream targets (Kawahara et al., 2009; Zhong et al., 2010; Mao et al., 2011; Pan et al., 2016).

### 2.2 Regulation of glucose homeostasis

The first insights into SIRT6’s roles in mammalian cells came from SIRT6 loss-of-function studies. SIRT6-deficient cells show increased sensitivity to DNA damage and genomic instability associated with defects in the base excision repair (BER) (Mostoslavsky et al., 2006). Moreover, *Sirt6* knockout (ko) mice are born normally but quickly exhibit characteristics of premature aging with profound lymphopenia, severe hypoglycemia, and greatly reduced serum levels of insulin-like growth factor 1 (IGF-1), ultimately leading to early death by around 4 weeks (Mostoslavsky et al., 2006). Subsequently, it was found that SIRT6 is a master regulator of glucose homeostasis by repressing multiple glycolytic genes and a major glucose transporter GLUT1 as a corepressor of HIF1α, a key modulator of nutrient and oxygen stress responses (Zhong et al., 2010). SIRT6 deficiency increases glucose uptake and thus triggers a nutrient stress response in the switch of glucose metabolism from mitochondrial respiration to glycolysis, indicating that the critical role of SIRT6 is to maintain an adequate glucose flux into the tricarboxylic acid cycle and oxidative phosphorylation for energy (i.e., ATP) production in the mitochondria under normal nutrient conditions (Zhong et al., 2010). As described later, this SIRT6 function is connected to the concept of the photoreceptor–RPE energy ecosystem and therefore has implications in retinal degenerative diseases such as retinitis pigmentosa (RP) and age-related macular degeneration (AMD) (Kanow et al., 2017; Leveillard et al., 2019; Hurley, 2021).

### 2.3 Enhancing DNA repair

SIRT6 activates PARP1 through its enzymatic activity as a mono-ADP-ribosylase to enhance DNA repair. In mammalian cells, SIRT6 is recruited to the sites of DNA double-strand breaks (DSBs) following exposure to oxidative stress and stimulates DSB repair via both nonhomologous end joining and homologous recombination (Mao et al., 2011). Mechanistically, SIRT6 physically associates with and mono-ADP-ribosylates PARP1 on lysine residue 521, thereby increasing PARP1 poly(ADP)-ribosylase activity and enhancing DSB repair under oxidative stress (Mao et al., 2011). SIRT6 is also involved in BER, as alluded to earlier in one of the initial SIRT6 studies showing defective BER and subsequent DNA damage and genomic instability in SIRT6-deficient cells (Mostoslavsky et al., 2006). Later studies have revealed that BER is also regulated by SIRT6 in a PARP1-depdendent manner, and that overexpression of SIRT6 rescues the decline of BER in aged fibroblasts (Xu et al., 2015). More recently, it has been shown that SIRT6 is also involved in the nucleotide excision repair (NER) pathway, which is responsible for removing bulky DNA adducts induced by UV irradiation or genotoxic chemicals (Geng et al., 2020). SIRT6-depleted cells by siRNA showed reduced NER efficiency of UV-irradiated GFP plasmids, whereas SIRT6-overexpressing cells had improved NER. Mechanistically, SIRT6 was found to interact with and deacetylate DNA damage-binding protein (DDB2), a major sensor initiating global genome NER, to promote DDB2 ubiquitination and segregation from chromatin, thus facilitating NER signaling cascades (Geng et al., 2020). The DNA damage response (DDR) is a highly orchestrated, multi-step process. SIRT6 is involved not only in DDR in different types of DNA damage as described above but also in different stages of DDR, from sensing DNA damage, relaxing chromatin, and recruiting a repair machinery, to repairing and restoring chromatin structure (Korotkov et al., 2021). For example, it has been reported that SIRT6 can directly recognize and bind to damaged DNA to serve as a DNA break sensor to subsequently activate downstream signaling for DSB repair (Onn et al., 2020). Interestingly, SIRT6 recognizes DNA breaks through the synergistic action with SIRT1 in that SIRT1 first deacetylates SIRT6 at residue K33, which is important for SIRT6 polymerization and mobilization toward DSBs (Meng et al., 2020). For DDR in eukaryotes, chromatin relaxation is required at DNA damage sites for accessibility of the DNA repair machinery. In this regard, SIRT6 interacts with and recruits CHD4, a core subunit of chromatin remodeling complex NuRD, to promote chromatin relaxation in response to DNA damage (Hou et al., 2020). Furthermore, SIRT6 interacts with and recruits various chromatin-related factors to DNA damage sites, thus promoting local chromatin accessibility, as well as restoring the original chromatin structure following DNA damage repair (Korotkov et al., 2021). Detailed molecular mechanisms by which SIRT6 promotes DDR at each step have been coming to light and will be further revealed in ongoing and future studies.

### 2.4 Antioxidant defense as a coactivator of NRF2

In response to oxidative stress, SIRT6 interacts with NRF2, a key transcriptional regulator of antioxidant defense, and upregulates antioxidant enzymes such as heme oxygenase 1 (HO-1, encoded by *HMOX1*) by acting as a coactivator of NRF2 in human mesenchymal stem cells (Pan et al., 2016). Although SIRT6 mainly functions as a gene silencer through its histone deacetylase activity, SIRT6 can also activate a subset of NRF2 target genes through mono-ADP-ribosylation of BAF170, a subunit of the ATP-dependent BAF chromatin remodeling complex (SWI/SNF complex in mammals), which gets recruited to the enhancer region of *HMOX1* and mediates the formation of a chromatin loop for transcriptional activation (Rezazadeh et al., 2019). Interestingly, mouse astrocytes that overexpress mutant human Cu-Zn superoxide dismutase 1 (*SOD1*), one of the genes associated with familial forms of amyotrophic lateral sclerosis (ALS), induce motor neuron death in coculture, but increasing the total NAD^+^ content or overexpressing SIRT6 in such astrocytes eliminates their toxicity to cocultured motor neurons (Harlan et al., 2019). Mechanistically, both increased NAD^+^ and SIRT6 overexpression result in NRF2 activation and upregulation of antioxidant proteins such as HO-1. Importantly, these observed neuroprotective effects require SIRT6 expression in astrocytes, suggesting a central, cell autonomous role for SIRT6 in abolishing the neurotoxicity in this ALS model (Harlan et al., 2019). It is notable that lamin A is an endogenous activator of SIRT6 and promotes SIRT6-mediated DNA repair (Ghosh et al., 2015), whereas NRF2 interacts with lamin A at the nuclear periphery (Kubben et al., 2016), suggesting that lamin A, SIRT6, and NRF2 may be functionally connected in aging-related networks (Gorbunova et al., 2016). Recent studies have further characterized the interactions between NRF2 and SIRT6. Using the yeast two-hybrid system, the NRF2-ECH homology (Neh) domains Neh1 and Neh3 have been identified as the key NRF2 protein regions necessary to interact with SIRT6 (Fu et al., 2023). Without the intact Neh1 and Neh3 domains, NRF2 lost its transcriptional activity to upregulate antioxidant genes. One of the key molecular steps in the NRF2 pathway by which SIRT6 controls NRF2 activity involves Kelch-like ECH-associated protein 1 (KEAP1), a negative regulator of NRF2. To activate NRF2, SIRT6 both suppresses KEAP1 expression and binds to NRF2 to inhibit interaction between NRF2 and KEAP1, stabilizing NRF2 levels (Kanwal et al., 2019). In *Sirt6* ^+/−^ mice, paraquat-induced oxidative stress caused more severe liver damage with a lower survival rate than in *Sirt6* ^+/+^ mice (Liu et al., 2023). In mouse tissues, SIRT6 deficiency decreased NRF2 protein levels and mRNA levels of its target genes; conversely, SIRT6 overexpression increased NRF2 protein levels. In addition, NRF2 protein degraded faster in *Sirt6* ^−/−^ mouse embryonic fibroblasts (MEFs) than in *Sirt6* ^+/+^ MEFs, and SIRT6 increased NRF2 protein accumulation in the nucleus. Mechanistically, SIRT6 bound to NRF2, and SIRT6 overexpression decreased KEAP1 binding to NRF2. These results suggest that SIRT6 also exerts antioxidant effects by increasing NRF2 protein levels through KEAP1-mediated regulation (Liu et al., 2023).

### 2.5 Repression of NF-κB target genes

SIRT6 interacts with the p65 subunit (also known as RELA) of NF-κB, a master regulator of inflammation, immune, and stress responses, resulting in its recruitment to the regulatory regions of NF-κB target genes and subsequent suppression of their expression by H3K9Ac deacetylation (Kawahara et al., 2009). Genome-wide chromatin immunoprecipitation-oligonucleotide microarray (ChIP-chip) assays have shown that SIRT6 dramatically changes its localization on mouse promoters in response to TNFα in a largely RELA-dependent manner, as it occupies more than half of its target genes only after addition of TNFα (Kawahara et al., 2011). Comparisons of global gene expression patterns in wild-type, *Sirt6* ^−/−^, and double *Sirt6* ^−/−^ *RelA* ^−/−^ mouse embryonic fibroblasts have revealed the epistatic relationship between SIRT6 and RELA that produces diverse, temporal patterns of gene expression, including joint control of several key genes in cell senescence and aging. These results suggest that a key output of NF-κB regulation of stress response and aging is likely mediated by SIRT6’s dynamic chromatin relocalization and its downstream effects on gene expression (Kawahara et al., 2011). However, Grimley et al. reported that SIRT6 overexpression had little effect on both TNFα-induced nuclear translocation of NF-κB and the expression of its target genes (Grimley et al., 2012). This discrepancy might be due to different experimental settings and cell types, which is discussed later in the context of the RPE. Given these findings were reported more than a decade ago, the role of SIRT6 in repressing NF-κB target genes have since been recognized in various physiological and pathological conditions. In osteoarthritis (OA), in which inflammation and aging play key roles, patients’ articular chondrocytes showed significantly decreased SIRT6 levels compare to those of healthy individuals (Wu et al., 2015). SIRT6 overexpression suppressed replicative senescence and decreased the expression of NF-κB target genes in human chondrocyte cultures treated with IL-1β. In an OA mouse model, intra-articular injection of lentivirus carrying *Sirt6* prevented mouse chondrocyte degeneration (Wu et al., 2015). As an *in vitro* OA model, primary mouse chondrocytes were treated with IL-1β, and SIRT6 activator ergothioneine (EGT) decreased the breakdown of collagen II and aggrecan and inhibited the increase of pro-inflammatory mediators in these chondrocytes (Wang Z. et al., 2023). Mechanistically, EGT inhibited NF-κB activity through SIRT6 activation, which in turn attenuated the IL-1β-induced inflammatory response. EGT’s inhibitory effects on OA was also observed in a mouse model of OA (Wang Z. et al., 2023). In mouse experimental colitis induced by dextran sulfate sodium salt, SIRT6 overexpression attenuated signs and histological damage in the colon, highlighting SIRT6’s protective effects on the colon (Xu et al., 2020). Mechanistically, SIRT6 overexpression inhibited the activation of NF-κB and c-Jun by regulating TAK1 signaling (Xu et al., 2020). In the context of vascular aging, which is one of the major factors in developing cardiovascular diseases, SIRT6 in the aortae was decreased in aged rats compared with young rats (Liu et al., 2021). In cultures of adventitial fibroblasts (Afs) from rat aortae, *Sirt6* knockdown promoted aging phenotypes, such as increased proliferation, collagen secretion, and migration. When exposed to angiotensin II (Ang II), Afs showed vascular aging phenotypes, along with decreased SIRT6 expression and activated NF-κB signaling. Importantly, these effects of Ang II were reduced by NF-κB pathway inhibitor. Mechanistically, *Sirt6* knockdown increased acetyl-NF-κB p65 (Lys310) levels and NF-κB transcriptional activity, suggesting that SIRT6 attenuates Ang II-induced vascular aging by inhibiting NF-κB activation (Liu et al., 2021). SIRT6 is also protective against hepatic injury. In a thioacetamide-induced acute liver failure mouse model, the SIRT6 activator UBCS039 attenuated liver damage, with decreased inflammatory responses and oxidative stress (Jiao et al., 2022). UBCS039 increased SIRT6 levels to inhibit inflammatory reactions by suppressing NF-κB signaling in this mouse model, as well as in lipopolysaccharide (LPS)-stimulated macrophage cultures. In parallel, upregulation of SIRT6 alleviated hepatic oxidative damage through the NRF2 pathway (Jiao et al., 2022). Additionally, SIRT6’s beneficial and protective effects have been reported in skin wound healing using MDL-800, a selective SIRT6 activator (Jiang et al., 2022), as well as in neurological recovery after intracerebral haemorrhage in a rat model (Cheng et al., 2023). In both cases, SIRT6’s effects were also mediated by reduced inflammatory response through suppression of NF-κB signaling.

### 2.6 Positive roles in lifespan and longevity

At the organismal level, SIRT6 has been associated with aging and longevity. As described above, *Sirt6* ^−/−^ mice are born normally but develop premature aging phenotypes, such as a loss of subcutaneous fat and kyphosis, eventually dying at around 4 weeks of age (Mostoslavsky et al., 2006). Importantly, these phenotypes of *Sirt6* ^−/−^ mice are rescued in *Sirt6* ^−/−^ *RelA* ^+/−^ mice by significantly reducing expression of NF-κB target genes, suggesting that hyperactive NF-κB signaling may be largely responsible for premature aging and the shortened lifespan observed in *Sirt6* ^−/−^ mice (Kawahara et al., 2009). Indeed, NF-κB was identified as a key activator of aging-related gene expression programs by comparing young and old mice (Adler et al., 2007). In this study, blocking NF-κB in skin cells of aged transgenic mice was found to reverse tissue-specific characteristics and gene expression patterns to those of younger mice, confirming the role of NF-κB in aging (Adler et al., 2007). Conversely, whole-body overexpression of SIRT6 in transgenic mice on the mixed-CB6 background (equal ratio of C57BL/6J and BALB/cOlaHsd) extended lifespan and healthspan of male mice, but not female mice (Kanfi et al., 2012). The authors found that male transgenic mice had lower levels of IGF-1 and higher levels of IGF-binding protein 1, as well as altered IGF-1 signaling, a key pathway in the regulation of lifespan (Kanfi et al., 2012). Yet, others have found that SIRT6 overexpression in the C57BL/6JOlaHsd background extended lifespan and reduced aging-associated frailty in both male and female mice (Roichman et al., 2021). Mechanistically, SIRT6 overexpression alleviates age-related decline of gluconeogenesis in the liver, *de novo* NAD^+^ synthesis, and glucose homeostasis for maintaining energy metabolism in old age (Roichman et al., 2021). Furthermore, long-lived rodent species have more efficient DNA DSB repair than short-lived rodents, and SIRT6’s activity to promote DSB repair is positively correlated with the lifespan of rodent species, underscoring the importance of DNA repair in longevity and highlighting another link between SIRT6 and lifespan (Tian et al., 2019). Using single cell whole-genome sequencing, the authors directly showed that bleomycin-induced somatic mutations in lung fibroblasts inversely correlated with maximum lifespan of rodent species, suggesting that longer-lived species have a higher capacity to process DNA damage to maintain genome integrity than shorter-lived species (Zhang et al., 2021). Beyond rodents, whole-genome sequencing of intestinal crypts from 16 mammalian species showed that the somatic mutation rate was inversely correlated with species lifespan, implying that somatic mutation rates may indeed contribute to aging (Cagan et al., 2022). Recently, a rare SIRT6 allele containing two linked substitutions (N308K and A313S) was identified by targeted sequencing of the SIRT6 locus in 450 Ashkenazi Jewish (AJ) centenarians and 550 AJ control individuals, with a higher allele frequency in people living beyond 100 years (thus named centSIRT6) (Simon et al., 2022). Compared with wild-type SIRT6, centSIRT6 more strongly suppressed LINE1 retrotransposons, more efficiently stimulated DNA DSB repair, and more robustly killed cultured cancer cells. Interestingly, centSIRT6 had weaker deacetylase activity but stronger mono-ADP ribosylase activity. This variant additionally interacted with Lamin A more strongly, which was correlated with enhanced ribosylation of Lamin A. These results suggest that enhanced SIRT6 function contributes to human longevity with better genome maintenance via increased mono-ADP ribosylase activity and stronger interaction with Lamin A (Simon et al., 2022). This centSIRT6 is also beneficial in preventing nonalcoholic liver disease such as steatohepatitis. Overexpression of centSIRT6 resulted in a dramatic change in the metabolome and secretome of immortalized human hepatocytes, with an increase of most amino acids, unsaturated fatty acids, and glycerophospholipids and decreased ceramide (Frohlich et al., 2023). Notably, recent studies have shown that the devastating phenotypes of *Sirt6* ^−/−^ mice could be alleviated by a high-fat diet (HFD). The lifespan of *Sirt6* ko mice was dramatically increased by HFD to 26 and 37 weeks in male and female mice, respectively, with a reversal of multi-organ atrophy, body weight loss, hypoglycemia, and premature aging (Li et al., 2020). Concomitantly, the global gene expression profiles were partially, but significantly, normalized in HFD-fed *Sirt6* ko mice. HFD attenuated excessive glucose uptake and glycolysis in skeletal muscle induced by SIRT6 deficiency through inhibition of insulin and IGF-1 signaling (Li et al., 2020). Furthermore, fecal microbiota transplantation from *Sirt6* ko mice into wild-type mice phenocopies the gut dysbiosis and premature aging observed in *Sirt6* ko mice. Conversely, when *Sirt6* ko mice received fecal microbiota from wild-type mice, their lifespan was extended (Xu et al., 2023). Antibiotics treatment reduced inflammation and cell senescence in *Sirt6* ko mice, suggesting that gut dysbiosis contributes to the premature aging phenotypes of *Sirt6* ko mice. Thus, gut microbial dysbiosis is likely causally linked to aging, which could explain the beneficial effect of HFD for correcting gut dysbiosis and alleviating premature aging (Xu et al., 2023).

### 2.7 Critical roles in early development

While SIRT6 has drawn great interest in the aging and longevity field, the protein also plays a critical role at the beginning of life during embryonic development. As described above, although *Sirt6* ko (null) mice on the 129SvJ background are born normally, they are smaller, develop a premature aging phenotype after 2 weeks old, and die around 4 weeks of age (Mostoslavsky et al., 2006). In contrast, SIRT6-null cynomolgus monkeys generated using a CRISPR–Cas9-based strategy show severe prenatal developmental retardation with significantly smaller brains and die a few hours after birth (Zhang et al., 2018). In these SIRT6-deficient monkeys, transcription of *H19*, a maternally expressed, imprinted, long non-coding RNA known to regulate fetal development, is significantly upregulated in all tissues, especially in the brain. This elevated *H19* expression is associated with the higher levels of H3K56Ac. To gain further insights into the roles of SIRT6 in the brain, the authors also generated *SIRT6*-null human embryonic stem cells (ESCs) using TALEN-mediated gene editing and differentiated them into neural progenitor cells (NPCs). These *SIRT6* ^*−/−*^ NPCs showed delayed neuronal differentiation accompanied by higher levels of H3K56Ac at the *H19* imprinting control region and more CTCF recruited to that region, along with upregulation of *H19*. In both models, SIRT6 rescued these defects (Zhang et al., 2018). Indeed, the impact of SIRT6 deficiency seems increasingly more devastating along the course of mammalian evolution (Naiman and Cohen, 2018). In humans, the homozygous *SIRT6* mutation c.187G>C; p.(Asp63His) (D63H) abolishes deacetylase and demyristoylase activities on H3K9 and causes severe neurodevelopmental and cardiac anomalies, leading to perinatal death before birth (Ferrer et al., 2018). Further analyses using mouse ESCs expressing the SIRT6 D63H mutant and human induced pluripotent stem cells (iPSCs) derived from D63H homozygous fetuses showed a failure to repress the expression of pluripotency genes like *Oct4*, *Sox2*, and *Nanog* after induction of differentiation, resulting in a failure to differentiate into embryoid bodies, cardiomyocytes, and NPCs (Ferrer et al., 2018). These results indicate that SIRT6 is a critical factor involved in repression of pluripotency genes in human early development. This persistent expression of pluripotency genes in *Sirt6* ko mouse ESCs increases production of 5-hydroxymethylcytosine (5hmC), particularly on neural genes, through upregulation of the ten-eleven translocation enzymes (TETs) that convert 5-methylcytosine into 5hmC. This finding can explain the neuroectoderm-skewed differentiation of SIRT6-deficient mouse ESCs (Etchegaray et al., 2015). Thus, SIRT6 functions as a chromatin regulator protecting the balance between pluripotency and differentiation of ESCs.

### 2.8 Regulation of stress granules

Despite the aforementioned SIRT6 functions all occurring within the nucleus, SIRT6 also plays a role in the cytoplasm. Two independent proteomics analyses of the SIRT6–interacting protein network identified Ras-GTPase-activating protein (SH3 domain)-binding protein 1 (G3BP1), a cytoplasmic protein and core component of stress granules (SGs), as one of the strongest interacting partners with SIRT6 (Simeoni et al., 2013; Miteva and Cristea, 2014). Subsequent studies have reported that SIRT6 rapidly translocates to the cytoplasm following heat shock and interacts with G3BP1 to regulate the assembly and disassembly of SGs in mouse embryonic fibroblasts (Tourriere et al., 2003; Jedrusik-Bode et al., 2013; Simeoni et al., 2013). *Sirt6*-deficient cells show disruption of SG assembly with increased cell death, suggesting SIRT6’s role in regulating SGs for cell survival (Jedrusik-Bode et al., 2013; Simeoni et al., 2013). SGs are cytoplasmic, membrane-less organelles formed by a process of liquid–liquid phase separation (LLPS) that contain RNA–protein complexes with translationally-stalled mRNAs, which are assembled in response to various stresses and disassembled during stress recovery (Protter and Parker, 2016; Wheeler et al., 2016; Wolozin and Ivanov, 2019). In response to increased free cytoplasmic RNAs, SGs are assembled by a network of protein–RNA interactions, in which G3BP1 is the central node and thus a critical molecular switch for LLPS (Guillen-Boixet et al., 2020; Yang et al., 2020). SGs are intended to be dynamic and transient during acute stresses; however, they can become more stable SGs under chronic aging and disease-associated stresses (Wolozin and Ivanov, 2019). Such persistent “pathological SGs” could act as a seed for aggregation of disease-related proteins in the pathophysiology of neurodegenerative diseases (Wolozin and Ivanov, 2019). Proteins undergoing LLPS such as RNA-binding proteins contain low-​complexity sequences or intrinsically disordered regions (IDRs) that can form the weak multivalent, macromolecular interactions essential for LLPS (Banani et al., 2017). Importantly, the C-terminus of SIRT6 is predicted to contain an IDR (Miteva and Cristea, 2014; Klein and Denu, 2020), supporting an intriguing idea that SIRT6 could indeed physically participate in phase separation of macromolecular condensates like SGs in the cytoplasm, as well as other structures in the nucleus. Further studies may reveal exciting new roles of SIRT6 in this largely unexplored area.

### 2.9 Regulation of macrophage polarization toward M2

Macrophages are known to have great plasticity and undergo phenotypic changes in response to a variety of stimulating signals such as microbes, damaged tissues, and cytokines (Biswas and Mantovani, 2010). Two distinct states of polarized macrophage activation have been recognized: 1) classically activated (M1) macrophages and 2) alternatively activated (M2) macrophages. However, there is substantial heterogeneity with intermediate and overlapping states of macrophage polarization, and transcriptome analysis revealed a spectrum of macrophage activation states with typical M1 and M2 phenotypes at the extremes of this spectrum (Biswas and Mantovani, 2010; Xue et al., 2014). LPS and interferon-γ (IFNγ) polarize macrophages toward the M1 phenotype that is regarded as pro-inflammatory, whereas interleukin 4 (IL-4) drives macrophages toward the M2 phenotype that is regarded as anti-inflammatory (Biswas and Mantovani, 2010). Recently, SIRT6 has been found to promote macrophage polarization toward the M2 phenotype, which can also contribute to SIRT6’s anti-inflammatory function. Adipocyte-specific *Sirt6* ko mice gain more body weight and fat mass than wild-type mice and show glucose intolerance and insulin resistance, with decreased M2 macrophages in the adipose tissue (Song et al., 2019). In this model, SIRT6 increased IL-4 expression in adipocytes to drive M2 macrophage polarization. Importantly, there was a negative correlation between the extent of obesity or insulin resistance and SIRT6 levels in human visceral fat tissues (Song et al., 2019). Myeloid cell-specific *Sirt6* ko mice showed delayed skin wound closure with less collagen deposition, suppressed angiogenesis, and reduced expression of wound healing-related genes compared with wild-type mice (Koo et al., 2019). These ko mice had increased infiltration of M1 macrophages with a decrease in M2 macrophages (Koo et al., 2019). In a mouse model of peripheral nerve injury and repair, SIRT6 was significantly upregulated in the crushed sciatic nerve, and upon shRNA silencing of SIRT6 the crushed nerve recovery was delayed (Zou et al., 2021). In cultured mouse macrophages, both the SIRT6 inhibitor OSS128167 and *Sirt6* shRNA knockdown reduced macrophage migration and phagocytic capacity. Importantly, SIRT6 inhibition led to macrophage polarization toward the M1 phenotype with decreased M2 macrophages through activation of NF-κB and TNFα signaling, and the SIRT6 activator UBCS039 induced a shift of macrophage polarization from the M1 to M2 phenotype (Zou et al., 2021). In OA, a similar relationship between SIRT6 and macrophage polarization was observed. OA patients with synovial inflammation had significantly lower serum IL-4 levels and decreased synovial M2 macrophages compared with control individuals (Chen J. et al., 2022). SIRT6 inhibition promoted the release of pro-inflammatory cytokines by synovial macrophages and induced M1 macrophage polarization with decreased M2 polarization *in vitro*. In contrast, SIRT6 overexpression alleviated OA in a mouse model, suggesting that SIRT6 could inhibit synovial inflammation (Chen J. et al., 2022). In a mouse model of postoperative cognitive dysfunction, caffeic acid phenethyl ester (CAPE), a powerful antioxidant and anti-inflammatory agent, suppressed oxidative stress and promoted the switch of microglia from the M1 type to the M2 type in the hippocampus, thereby ameliorating cognitive impairment caused by anesthesia and surgery through upregulation of SIRT6 and NRF2 (Wang Y. et al., 2023). Moreover, CAPE reduced generation of reactive oxygen species (ROS) and promoted M2 polarization in H_2_O_2_-treated BV2 mouse microglial cells, which was attenuated by OSS128167 (Wang Y. et al., 2023).

### 2.10 Promoting mitochondrial function

Among seven sirtuins (SIRT1-7), while SIRT3, 4, and 5 are localized to mitochondria, SIRT6 is primarily a nuclear protein (Chang et al., 2020). However, SIRT6 has been found to play an important role in mitochondrial biology, including biogenesis, fusion–fission dynamics, and function. *Sirt6* knockdown in MIN6 mouse pancreatic beta cells resulted in a significant decrease in glucose-stimulated insulin secretion (GSIS) and mitochondrial oxygen consumption rates (Xiong et al., 2016). In *in vivo* studies, pancreatic beta cell-specific *Sirt6* ko mice showed a ∼50% decrease in GSIS and lower ATP levels in the islets compared with wild-type mice. In addition, the *Sirt6* ko islets displayed multiple signs of mitochondrial abnormalities, such as attenuated calcium dynamics, increased mitochondrial damage, and decreased mitochondrial complex levels. These findings suggest that SIRT6 is important for GSIS from pancreatic beta cells, and that SIRT6 activation may improve insulin secretion in diabetes (Xiong et al., 2016). Notably, while SIRT6 and SIRT3 have a myriad of functions that provide health benefits by themselves, it has been found that SIRT6 and SIRT3 also regulate each other’s activity and protect the heart from developing diabetic cardiomyopathy (Kanwal et al., 2019). In cardiomyocytes treated with palmitate as well as in the hearts of obese, diabetic mice fed with a high-fat, high-sucrose (HF-HS) diet, expression of both SIRT6 and SIRT3 decreased. Systemic SIRT6 overexpression protected mice from developing obesity and insulin resistance when fed with the HF-HS diet, and the hearts of these mice were also protected from mitochondrial fragmentation and SIRT3 downregulation. Mechanistically, SIRT3 preserves SIRT6 levels by reducing oxidative stress, whereas SIRT6 maintains SIRT3 levels through NRF2-mediated *Sirt3* transcription. To activate NRF2, SIRT6 both suppresses expression of KEAP1, a negative regulator of NRF2, and inhibits interaction of KEAP1 with NRF2 by competitively binding to NRF2, leading to stable NRF2 levels in cardiomyocytes as described earlier (Kanwal et al., 2019). The role of SIRT6 in regulating mitochondrial function was also shown in a mouse model of alcoholic cardiomyopathy using polydatin, a compound in traditional Chinese medicine (Yu et al., 2022). Polydatin treatment improved cardiac function, reduced myocardial fibrosis, inhibited mitochondrial fission mediated by dynamin-related protein 1 (DRP1), and activated PINK1–Parkin-dependent mitophagy in the myocardium. Importantly, SIRT6 knockdown by shRNA abolished these beneficial effects of polydatin, while SIRT6 overexpression mimicked the effects of polydatin treatment. Mechanistically, polydatin activated SIRT6–AMPK signaling to modulate mitochondrial dynamics and mitophagy, which preserved mitochondrial function and reduced oxidative stress (Yu et al., 2022). Decreased SIRT6 levels and mitochondrial fission were also observed in podocytes in Ang II-induced kidney injury in mice, and mitochondrial fragmentation and apoptosis were aggravated in podocyte-specific *Sirt6* ko mice (Chen Z. et al., 2022). Likewise, in cultured human podocytes treated with Ang II, mitochondrial fission was exacerbated by SIRT6 knockdown but ameliorated by SIRT6 overexpression. Mechanistically, SIRT6 deficiency promoted DRP1 phosphorylation by increasing expression of RHO-associated coiled coil-containing protein kinase 1 (ROCK1). These results show that SIRT6 is a crucial factor that protects against Ang II-induced mitochondrial fission and apoptosis in podocytes via the ROCK1-DRP1 signaling pathway (Chen Z. et al., 2022). Most recently, the brain has been added to the list of organs in which SIRT6 plays a crucial role in maintaining mitochondrial homeostasis and function. Transcriptomic analysis of brain tissue from wild-type and brain-specific *Sirt6* ko mice revealed that SIRT6 deficiency led to mitochondrial dysfunction with a global downregulation of mitochondria-related genes (Smirnov et al., 2023). Consistent with these results, metabolomic comparisons of wild-type and *Sirt6* ko mouse ESCs and SH-SY5Y neuroblastoma cells showed substantial differences in mitochondrial metabolite abundance in SIRT6-deficient cells, which was accompanied with elevated ROS production, decreased mitochondrial number, and reduced membrane potential (Smirnov et al., 2023). In these models, SIRT6 was shown to interact with transcription factor YY1 for coordinated regulation of mitochondrial gene expression, with the mitochondrial sirtuins SIRT3 and SIRT4 among the SIRT6 targets. Importantly, signs of mitochondrial dysfunction observed in SIRT6-deficient brains were also found in the aged brain and especially in the brain with neurodegenerative diseases, such as Alzheimer’s, Parkinson’s, Huntington’s, and ALS. These results suggest that SIRT6 is a central regulator of mitochondrial activity in the brain and that preserving SIRT6 level and activity in the aging brain could provide a therapeutic or preventive opportunity for neurodegenerative diseases by restoring mitochondrial homeostasis (Smirnov et al., 2023).

## 3 SIRT6 in the retina

**FIGURE 2:**
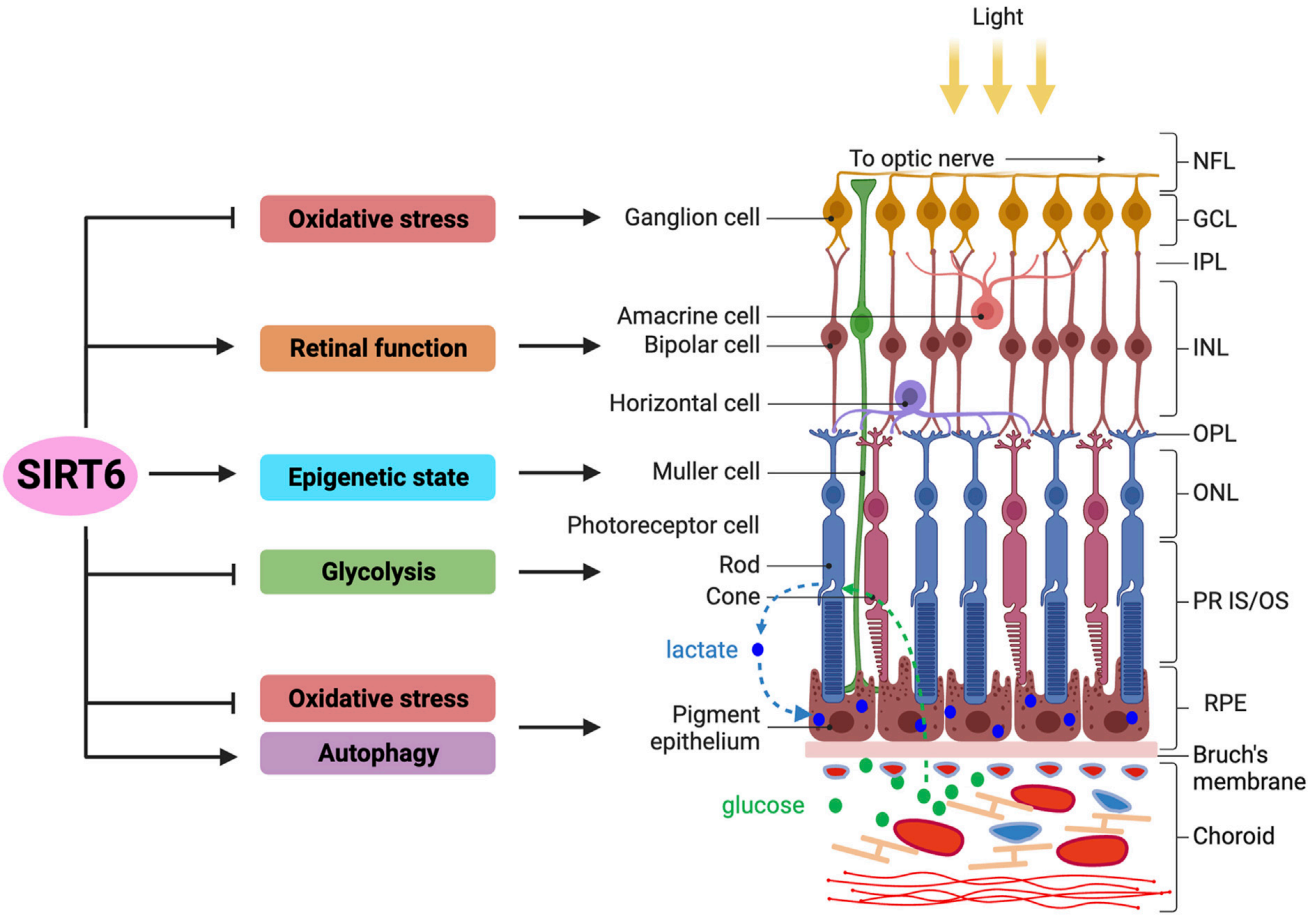
Overview of SIRT6’s functions in the retina and RPE. The functions of SIRT6 in the retina and RPE reported thus far are summarized (*left panel*), in connection with specific cell types or layers in these tissues (*right panel*). The retina of vertebrate eyes has three nuclear layers: outer nuclear layer (ONL), inner nuclear layer (INL), and ganglion cell layer (GCL). The RPE is a monolayer of epithelial cells located between the retina and the choroid containing blood vessels. Functionally, the retina initiates visual signals in photoreceptor cells by converting photons of light to electrical signals, which are then transferred to the brain. With multiple specialized functions, the RPE supports the survival and functions of retinal photoreceptors. Photoreceptor cells uptake glucose as an energy source from the choroid through the RPE and convert it to lactate, which is then exported to RPE cells as their energy source, forming a symbiotic relationship between these two cell types in energy metabolism. Regarding the functions of SIRT6 in the retina, studies show that SIRT6 protects rat primary retinal ganglion cells from oxidative stress through an NRF2-dependent pathway. Studies using *Sirt6* knockout mice show that SIRT6 is required for normal retinal function through its regulation of glucose and glutamate metabolism, especially in the INL. In addition, SIRT6 plays a protective role in early neurodegenerative events in mouse models of diabetic retinopathy, particularly by controlling the epigenetic state in Müller cells. Lastly, SIRT6 regulates energy metabolism by suppressing glycolysis that affects photoreceptor cell survival in a mouse model of retinitis pigmentosa, in which SIRT6 ablation attenuates photoreceptor degeneration by increasing aerobic glycolysis. In the RPE, SIRT6 has been shown to activate autophagy to modulate inflammation in culture cells, as well as protect RPE cells from oxidative stress in mice. Abbreviations: NFL, nerve fiber layer; GCL, ganglion cell layer; IPL, inner plexiform layer; INL, inner nuclear layer; OPL, outer plexiform layer; ONL, outer nuclear layer; PR IS/OS, photoreceptor inner segment/outer segment; RPE, retinal pigment epithelium.

The functions of SIRT6 in the retina reported thus far are described below and summarized in connection with specific cell types or layers in Figure 2.

### 3.1 Sirtuin gene expression in the retina

All seven sirtuin genes have been found to be ubiquitously expressed in a wide variety of human tissues (Michishita et al., 2005). Among these, higher levels of all sirtuin mRNAs were found in brain tissue, with *SIRT1* and *SIRT6* being highly expressed particularly in the fetal brain (Michishita et al., 2005). Although this study did not include eye tissues, a separate genome-wide transcriptome analysis of 79 human and 61 mouse tissues that included the retina showed that sirtuins are expressed in tissues throughout the body at largely comparable levels (BioGPS http://biogps.org) (Su et al., 2004; Wu et al., 2009; Wu et al., 2016). Another study of mouse sirtuin expression reported that all seven sirtuin genes are highly expressed in the retina compared to other tissues (Ban et al., 2013). Interestingly, in this study, mRNA levels of all sirtuin genes except *Sirt6* showed daily variations in the 12-h light/12-h dark cycle with increased levels in the dark phase, whereas *Sirt6* mRNA levels remained constant regardless of the light or dark conditions (Ban et al., 2013).

### 3.2 Effect on adult retinal function

Silberman et al. reported that SIRT6 is highly expressed in adult mouse retina, and general *Sirt6* ablation in mice significantly increased the levels of H3K9Ac and H3K56Ac in the retina, as expected given the protein’s function as a histone deacetylase (Silberman et al., 2014). The authors found that while *Sirt6* ko mice have normal retinal histology, these mice have severely impaired electroretinograms at all conditions tested at 20 days old, suggesting that SIRT6 is required for normal retinal function. At the molecular level, SIRT6 deficiency resulted in upregulation of glycolytic genes and GLUT1 (encoded by *Slc2a1*) expression, as well as downregulation of glutamate receptors, including glutamate metabotropic receptor 6 (*Grm6*) that is expressed in ON bipolar cells (Silberman et al., 2014). These changes were accompanied by increased numbers of TUNEL-positive cells, implying apoptotic cell death in the inner nuclear layer (Silberman et al., 2014). Thus, SIRT6’s role in maintaining normal retinal function is likely exerted through its regulation of energy (glucose) metabolism and glutamate metabolism at retinal synapses. Yet, a conflicting study using an inducible conditional ko (cko) of *Sirt6* in rod photoreceptors did not find any electroretinogram abnormalities or changes in retinal histology after induction of SIRT6 deficiency by tamoxifen (Zhang et al., 2016). Taken together, these contradictory findings suggest that the precise nature of SIRT6’s role in modulating adult retinal function requires further investigation.

### 3.3 SIRT6 inhibition attenuates retinal degeneration

RP is a genetically diverse group of inherited retinal diseases that ultimately cause blindness from the progressive degeneration of rod photoreceptor cells, followed by secondary cone cell death (Hartong et al., 2006). Mutations in non-syndromic RP have been identified in over 80 genes (Retinal Information Network, RetNet https://web.sph.uth.edu/RetNet/), including the widely-studied cGMP phosphodiesterase 6 (*PDE6*) genes associated with autosomal recessive RP (arRP) (Verbakel et al., 2018). In a mouse model of arRP with a homozygous hypomorphic mutation (H620Q) in the gene encoding the β subunit of PDE6 (*Pde6b*), photoreceptor degeneration begins at around 2–3 weeks of age, and nearly all photoreceptors are lost by 7–8 weeks of age (Hart et al., 2005; Davis et al., 2008). Zhang et al. combined this arRP mouse model with a tamoxifen-inducible *Sirt6* cko in rod photoreceptors (*Sirt6* ^*−/−*^ *Pde6b* ^*H620Q/H620Q*^ *Pde6g* ^*CreERT2*^) to test whether increased aerobic glycolysis by SIRT6 ablation could preserve rod and cone photoreceptors (Zhang et al., 2016). In this study, SIRT6 deficiency slowed photoreceptor degeneration both histologically and functionally, with better preserved outer nuclear layer (ONL) and photoreceptor outer segment (OS) length and significantly larger b-waves in scotopic, photopic, and mixed electroretinogram responses. Biochemical analyses showed that rod-specific SIRT6 ablation increased glycolytic flux and reprogrammed rod photoreceptors into a persistently glycolytic state, leading to the accumulation of glycolytic intermediates, with a dramatic increase in the amount of pyruvate and lactate (Zhang et al., 2016). In addition to the *Sirt6* cko strategy, *Sirt6* ablation using an adeno-associated virus (AAV)–*Sirt6* shRNA vector in the same arRP mouse model also rescued both rod and cone photoreceptors, as seen by histology and electroretinography. These results highlight the therapeutic potential of reprogramming metabolism to increase glycolysis, which may be applicable to a wide range of retinal degenerations (Zhang et al., 2016).

From the viewpoint of physiological roles of SIRT6, it should be noted that SIRT6 inhibition is beneficial in the case of metabolic reprogramming to attenuate retinal degeneration, while many favorable effects of SIRT6 result from its activation or overexpression in other contexts such as inflammation, oxidative stress, and DNA repair, as described earlier. This unusually negative effect of SIRT6 on cell survival in the arRP mouse model is likely because of the unique situation of energy (glucose) metabolism in photoreceptor cells. Photoreceptors uptake glucose from the choroid through RPE cells and utilize this glucose as an energy source in both mitochondrial respiration and aerobic glycolysis that produces lactate, which is then exported back to RPE cells as their energy source in mitochondrial respiration (Kanow et al., 2017; Hurley, 2021). Such active aerobic glycolysis (also known as the “Warburg effect”) under normal conditions is unique to cells that require increased synthesis of macromolecules, like phospholipids for generating new lipid membranes, such as continuously proliferating cells (i.e., tumor cells) and photoreceptor cells that renew 10% of their outer segments daily (Vander Heiden et al., 2009; Leveillard et al., 2019; Hurley, 2021). Aerobic glycolysis provides essential molecules for phospholipid synthesis, such as glycerol produced from glycolytic intermediates and NADPH produced through the pentose phosphate pathway, a sidestep pathway at the beginning of glycolysis (Venkatesh et al., 2015; Leveillard et al., 2019; Viegas and Neuhauss, 2021). In summary, SIRT6 acts against the needs of glycolysis in photoreceptors by repressing glycolytic genes and the glucose transporter GLUT1; thus, SIRT6 inhibition can provide beneficial effects in RP mouse models.

### 3.4 Protection of RGCs from oxidative stress

Section 2.4 described how SIRT6 and NRF2 are often linked in antioxidant defense. One important connection between the two proteins involves BTB domain and CNC homolog 1 (BACH1), a transcriptional repressor that competes with NRF2 for binding to antioxidant response elements (AREs) in oxidative stress-response genes (Dhakshinamoorthy et al., 2005). Hepatocyte-specific SIRT6 deletion in mice downregulates NRF2 and upregulates BACH1 in the liver (Ka et al., 2017). Conversely, SIRT6 induces detachment of BACH1 from AREs in the *HO-1* gene promoter and promotes binding of NRF2 to the AREs in response to oxidative stress in HepG2 human hepatoma cells (Ka et al., 2017). In the retina, SIRT6 overexpression increases cell survival of rat primary retinal ganglion cells (RGCs) treated with H_2_O_2_ in culture and decreases their apoptotic cell death and production of ROS (Yu et al., 2019). Conversely, SIRT6 knockdown using siRNA has shown the opposite effect. Mechanistically, SIRT6 overexpression results in NRF2 accumulation in the nucleus, which leads to increased transcriptional activity of AREs, along with simultaneous inhibition of BACH1 expression (Yu et al., 2019). As evidence for their mechanistic roles, either BACH1 overexpression or NRF2 knockdown partially abolishes SIRT6’s protective effects against H_2_O_2_-induced oxidative damage. Thus, SIRT6 protects primary RGCs from H_2_O_2_-induced oxidative stress through the SIRT6–BACH1–NRF2/ARE axis (Yu et al., 2019).

### 3.5 Role in diabetic retinopathy

A role for SIRT6 in diabetic retinopathy (DR), one of the common microvascular complications of diabetes mellitus, has also been suggested during early neurodegenerative events that precede any detectable vascular changes. In non-obese diabetic (NOD) mice that naturally develop type 1 diabetes, high glucose (>200 mg/dL) leads to an increase of vascular endothelial growth factor (VEGF) and a decrease of neuroprotective brain-derived neurotrophic factor (BDNF), along with reduced levels of SIRT6 protein and increased levels of H3K9Ac and H3K56Ac in the retina (Zorrilla-Zubilete et al., 2018). Importantly, the retinas of central nervous system (CNS)-specific *Sirt6* cko mice with Nestin promoter-driven Cre recombinase (Nes–Cre–SIRT6^−/−^) show thinning of retinal layers with increased levels of VEGF and decreased levels of BDNF similar to the NOD mice, suggesting an ongoing neurodegenerative process (Zorrilla-Zubilete et al., 2018). In these *Sirt6* cko mice, increased levels of H3K9Ac and H3K56Ac are detected particularly in the inner nuclear layer where interneurons and Müller cells reside. Cultured primary Müller cells exposed to high glucose recapitulate the expression changes of SIRT6, H3K56Ac, VEGF, and BDNF observed in the NOD mice, and such changes are reversed with SIRT6 overexpression. These findings suggest that epigenetic deregulation may underlie early neurodegenerative events in DR prior to vascular changes (Zorrilla-Zubilete et al., 2018). In addition, Müller cells cultured with high glucose show increased expression of the pluripotent factor SOX9 and decreased levels of SIRT6 with higher levels of H3K9Ac, and siRNA-mediated *SIRT6* knockdown also increases SOX9 levels (Sanhueza Salas et al., 2021). The combination of high glucose and oxidative stress reduced levels of glutamine synthetase, a Müller cell marker, and increased the migration capacity of the cells, suggesting that these conditions can, to some extent, induce dedifferentiation with migration ability (Sanhueza Salas et al., 2021). Furthermore, gene expression profiling and Gene Ontology enrichment analysis of Müller cells from a streptozotocin-induced rat model of diabetes showed enrichment of genes associated with glucose metabolism, cell migration, development, and pluripotency, many of which were related to SIRT6 functions (Gerhardinger et al., 2005; Sanhueza Salas et al., 2021). Thus, SIRT6 may indeed be involved in DR, particularly through its role in controlling the epigenetic state of Müller cells and thereby changing the physiological functions of the cells.

## 4 SIRT6 in the RPE

**FIGURE 3:**
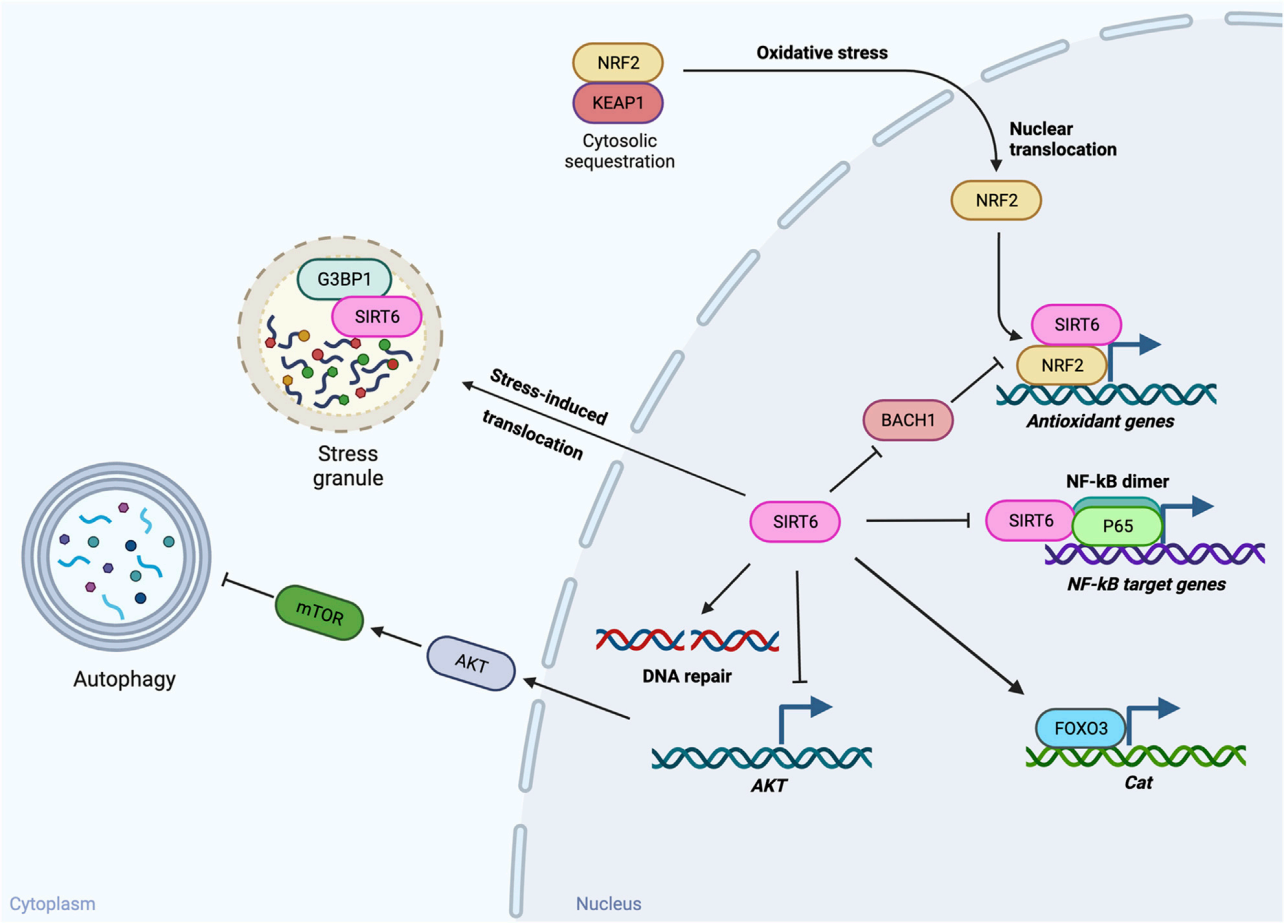
Potential mechanisms of SIRT6’s functions in autophagy and oxidative stress. SIRT6 has been found to play a role in autophagy and oxidative stress in the RPE. The possible mechanisms by which SIRT6 activates autophagy and protects against oxidative stress summarized here are based on studies in both RPE and non-RPE cells. In response to inflammatory stimuli, SIRT6 activates autophagy in cultured RPE cells. Although the effects of autophagy on inflammation in RPE cells are still inconclusive, multiple reports in other tissues support the beneficial effects of SIRT6-induced autophagy. One possible mechanism by which SIRT6 induces autophagy is suppression of *AKT* expression and thereby reduction of AKT–mTOR signaling that inhibits autophagy. Against oxidative stress, SIRT6 can protect cells through multiple pathways. SIRT6 overexpression protects the RPE from oxidative stress by preserving expression of catalase (encoded by *Cat*) in mice, possibly through FOXO3 activation. Based on studies with other tissues, SIRT6 has been shown to protect cells from oxidative stress through suppression of NF-κB target genes by interacting with p65 of NF-κB, upregulation of antioxidant genes by acting as a coactivator of NRF2 and/or suppressing expression of BACH1 that competes with NRF2 for binding to antioxidant response elements, and stimulation of DNA repair. In addition, SIRT6 can also manage stresses by regulating stress granules (SGs) through interaction with G3BP1, a core component of SGs, in the cytoplasm.

Both *Sirt6* mRNA and SIRT6 protein are readily detectable in mouse RPE samples without choroid by RT-qPCR and Western blotting, respectively (Yang et al., 2023). Yet, in contrast to the retina, studies of SIRT6 in the RPE are still scarce, with only three PubMed entries as of 10/10/2023. These include two papers related to autophagy (Feng et al., 2018; Liu and Liu, 2020) and our study related to oxidative stress (Yang et al., 2023) (Figure 2). We describe these studies and discuss implications of their findings below. The possible mechanisms by which SIRT6 activates autophagy and protects against oxidative stress are summarized from studies in both RPE and non-RPE cells in Figure 3.

### 4.1 Activation of autophagy

#### 4.1.1 SIRT6 activates autophagy (general and other tissues)

Autophagy is a lysosome-mediated intracellular degradation system for cytoplasmic components and plays an important role in a variety of physiological and pathophysiological processes, such as adaptation to nutrient deprivation, clearance of intracellular proteins and organelles, development, anti-aging, elimination of microorganisms, cell death, tumor suppression, and immune response (Mizushima, 2007; Rubinsztein et al., 2011; Klionsky et al., 2021). Autophagy can be classified into two types: 1) “induced autophagy” that is initiated during starvation to produce amino acids and 2) “basal autophagy” that continuously occurs in cells for the homeostatic turnover of cytoplasmic components (Mizushima, 2007; Lahiri et al., 2019). Given such important physiological roles in diverse homeostatic processes, it is no wonder that autophagy defects are associated with pathological changes across many organ systems, including the heart, liver, kidney, lung, brain, and eye, that result in neurodegenerative diseases, metabolic disorders, cancers, and many other human diseases (Klionsky et al., 2021). The role of SIRT6 in autophagy was first investigated in the context of cellular senescence in chronic obstructive pulmonary disease (COPD) (Takasaka et al., 2014). This study found that autophagy controls cellular senescence by eliminating damaged cellular components, which is negatively regulated by IGF-AKT signaling through the mammalian target of rapamycin (mTOR). Lung tissues from COPD patients showed potential signs of decreased autophagic capacity, which may contribute to cigarette smoke-induced senescence of human bronchial epithelial cells (HBECs). These COPD lung tissues also showed decreased levels of SIRT6 expression (Takasaka et al., 2014), and SIRT6 was also downregulated in cultured HBECs exposed to cigarette smoke extract (CSE). Importantly, SIRT6 overexpression inhibited CSE-induced HBEC senescence, whereas SIRT6 knockdown and a mutant SIRT6 (H133Y) lacking histone deacetylase activity exacerbated HBEC senescence. Mechanistically, while SIRT6 overexpression induced autophagy by reducing IGF-AKT-mTOR signaling, both SIRT6 knockdown and the mutant SIRT6 (H133Y) inhibited autophagy. SIRT6-induced autophagy was also reported as one of SIRT6’s multiple protective functions against renal podocyte injury and proteinuria (Liu et al., 2017). In this study, renal biopsies from patients with podocytopathies showed decreased levels of SIRT6, which correlated with glomerular function. Podocyte-specific *Sirt6* deficiency exacerbated podocyte injury in two mouse models, diabetic nephropathy and Adriamycin-induced nephropathy. SIRT6 overexpression by lentivirus vectors in the kidney significantly ameliorated renal injury of the Adriamycin-treated *Sirt6* cko mice. Further molecular analysis showed that SIRT6 decreased *Notch1* and *Notch4* transcription through H3K9 deacetylation in the promoter region of these genes, reducing Notch signaling and thereby restoring autophagy (Liu et al., 2017). Another example of SIRT6’s role in autophagy is observed in self-recovery in an LPS-induced mouse model of acute kidney injury. In this model, TNFα, IL-6, SIRT6, and LC3B-II/LC3B-I were initially increased 12 h after LPS injection, followed by a significant decrease at 48 h (Zhang et al., 2019). SIRT6 overexpression decreased the secretion of TNFα and IL-6, inhibited apoptosis induced by LPS, and promoted autophagy in HK-2 human kidney tubular epithelial cells, suggesting that the initial increase in SIRT6 levels might be connected to the repair of LPS-induced kidney damage (Zhang et al., 2019).

#### 4.1.2 SIRT6 activates autophagy in RPE cells

Thus far, two publications have described the role of SIRT6 in autophagy in connection with inflammation. In one study by Feng et al., RPE cells of aged mice accumulated subretinal deposition of amyloid-β (Aβ) and concomitantly upregulated the expression of SIRT6 and autophagic markers. This upregulation was also observed in Aβ-stimulated cultured RPE cells (Feng et al., 2018). Gain and loss-of-function analyses showed that SIRT6 activated autophagy and revealed that inhibition of autophagy attenuated Aβ-stimulated inflammatory response of RPE cells, suggesting that autophagy activated by SIRT6 promote Aβ-induced inflammation (Feng et al., 2018). Interestingly, this inflammation-promoting effect is opposite to the protective effect of autophagy against A2E-induced inflammation in ARPE19 cells (Zhang et al., 2015). Consistent with a protective role for autophagy, SIRT6 inhibited LPS-induced inflammation, oxidative stress, and apoptosis, partly by enhancing autophagy in ARPE19 cells (Liu and Liu, 2020). Thus, SIRT6 activates autophagy, but its effects on inflammation seem to depend on the types of triggers for the RPE inflammatory response. Although the outcomes of SIRT6-mediated autophagy in the RPE need further clarification, multiple reports support the beneficial effects of the SIRT6-autophagy axis in other tissues, including HBECs, kidney podocytes, and renal tubular epithelial cells, as described above (Takasaka et al., 2014; Liu et al., 2017; Zhang et al., 2019). Considering the defects of autophagy as one of the possible mechanisms for developing AMD, particularly the appearance of drusen deposits in dry AMD (Mitter et al., 2014; Golestaneh et al., 2017; Lakkaraju et al., 2020; Kaarniranta et al., 2023), SIRT6 could be a potential preventive and/or therapeutic target for AMD through activation of autophagy. Further studies are warranted in this area.

### 4.2 Protection against oxidative stress

#### 4.2.1 SIRT6 protects against oxidative stress (general and other tissues)

While SIRT6’s protective effect against oxidative stress has been previously documented in other tissues (Pan et al., 2016; Wang et al., 2016; Ka et al., 2017; Kim et al., 2019; Yu et al., 2019), studies of such roles in the RPE were lacking. However, we have recently found that SIRT6 indeed protects mouse RPE from sodium iodate (NaIO_3_)-induced oxidative stress *in vivo* (Yang et al., 2023). Considering that SIRT6 is a multifunctional protein, the mechanisms of the protective effects against oxidative stress could also be numerous, including suppression of NF-κB activity, upregulation of antioxidant genes through NRF2, stimulation of DNA repair, and regulation of SGs, depending on the sources and severity of oxidative stress, affected cell types, and many other biological factors. In addition to the NRF2-dependent mechanisms described in the earlier Section 2.4, reports have described NRF2-independent mechanisms by which SIRT6 protects against oxidative stress. One such mechanism seems to be SIRT6’s regulation of catalase expression, which is discussed in the later Section 4.3 (SIRT6, NRF2, and catalase).

#### 4.2.2 Translocation of SIRT6 from the nucleus to the cytoplasm in response to stress

Our recent studies showed that SIRT6 rapidly translocates from the nucleus to the cytoplasm of RPE cells in mice in response to NaIO_3_-induced oxidative stress, leading to acute SIRT6 depletion in the nucleus, particularly in the central region (Yang et al., 2023). Concomitantly, SIRT6 colocalized with G3BP1 (Kang et al., 2021), a key component of SGs, as previously reported in mouse embryonic fibroblasts following heat shock (Jedrusik-Bode et al., 2013; Simeoni et al., 2013). This response was fast, to the substantial degree that it was easily detected by immunofluorescence with anti-SIRT6 antibodies, and was also observed *ex vivo* in response to physical stress by peeling off the retina. Following translocation, SIRT6 remained in the cytoplasm and did not return to the nucleus even after G3BP1 aggregates disappeared six or 12 h later, resulting in longer SIRT6 depletion in the nucleus (Yang et al., 2023). Although regulation of SGs in the cytoplasm is an important stress response, this phenomenon presents a dilemma because SIRT6 is generally a nuclear protein with many nuclear-centric functions.

#### 4.2.3 SIRT6 overexpression in the nucleus protects RPE from oxidative stress

Since most of SIRT6’s functions are executed in the nucleus, the cytoplasmic localization following stress likely hinders its critical functions that are needed to combat oxidative stress in the nucleus. To overcome this nuclear SIRT6 depletion, a transgenic mouse line was generated using the mutant estrogen receptor ER^T2^ combined with the RPE-preferential human *BEST1* promoter for inducible SIRT6 overexpression in the nucleus. In these transgenic mice, tamoxifen-induced SIRT6 was retained in the nucleus and protected RPE cells from NaIO_3_-induced oxidative stress, resulting in milder RPE damage (Yang et al., 2023). These results suggest that in response to oxidative stress, SIRT6 would be needed for both regulating SG formation in the cytoplasm and controlling gene expression as an epigenetic chromatin regulator in the nucleus.

As described earlier, SIRT6 interacts with p65 of NF-κB to suppress NF-κB target genes by H3K9 deacetylation (Kawahara et al., 2009; Kawahara et al., 2011); therefore, the suppression of NF-κB targets is suspected as one mechanism of SIRT6’s protective effects against oxidative stress. Indeed, an IKKβ inhibitor, an upstream inhibitor of the NF-κB signaling pathway, protected mouse RPE from NaIO_3_-induced oxidative stress in the same manner as transgenic mice with SIRT6 overexpression (Yang et al., 2021). However, gene expression analyses showed that SIRT6 overexpression did not result in significant suppression of NF-κB target genes upregulated by NaIO_3_ in the transgenic mice (Yang et al., 2023). Instead, SIRT6 overexpression in the nucleus partially preserved expression levels of catalase (encoded by *Cat*), anti-oxidant enzyme that degrades H_2_O_2_ to H_2_O and O_2_ (Ho et al., 2004; Goyal and Basak, 2010). Thus, the protective effects of SIRT6 overexpression against oxidative stress are unlikely mediated by suppressing NF-κB targets in the NaIO_3_ mouse model (Yang et al., 2023). Since SIRT6 is a multi-functional protein, as this review has highlighted, the final outcomes of SIRT6’s actions are likely produced by a combination of several pathways, and the contribution of each pathway may be different depending on experimental settings.

### 4.3 SIRT6, NRF2, and catalase

#### 4.3.1 Role of catalase in the RPE

In the aforementioned SIRT6 overexpression studies in transgenic mice, mRNA levels were correlated between the *SIRT6* transgene and endogenous *Cat*, as well as between *Cat* and the RPE marker genes *Otx2* and *Rpe65* (Yang et al., 2023). Additionally, our earlier study revealed that the RPE in female mice was more susceptible to NaIO_3_ than the RPE in male mice, and that the mRNA levels of RPE markers, particularly *Otx2* and *Rlbp1*, were well correlated with those of *Cat* (Yang et al., 2021). These findings call attention to catalase and its regulation and relationship with SIRT6. A protective role of catalase in the RPE against retinal oxidative stress has been previously reported, where adenovirus-mediated transduction of catalase into mouse RPE protected the neighboring photoreceptors from oxidative stress in a light damage model (Rex et al., 2004). In this work, the authors speculated that because H_2_O_2_ can diffuse across membranes, overexpression of catalase in RPE cells could degrade H_2_O_2_ produced by light-induced oxidative stress in photoreceptors (Rex et al., 2004). Therefore, it is likely that the preserved expression of catalase is one mechanism by which SIRT6 overexpression protects the RPE against NaIO_3_-induced oxidative stress. Interesting parallels of SIRT6 and catalase have been reported, where both general overexpression of SIRT6 and mitochondrial overexpression of catalase have been found to extend mouse lifespan (Schriner et al., 2005; Kanfi et al., 2012).

#### 4.3.2 SIRT6 increases catalase expression

Although NRF2 plays a key role in regulating many antioxidant enzymes through its binding to AREs in gene promoters to induce their expression (Ma, 2013; Tonelli et al., 2018), it is still controversial whether NRF2 plays a role in the regulation of *Cat* expression because there are no AREs present in the regulatory region of *Cat* (Glorieux et al., 2015). This situation implies that there may be other mechanisms by which SIRT6 upregulates catalase to protect mouse RPE against oxidative stress. Interestingly, myocardial damage caused by ischemia/reperfusion (I/R) injury was more severe in *Sirt6* ^+/−^ heterozygous ko mice, but this deleterious effect was reversed by restoration of SIRT6 expression by a cardiac injection of adenovirus carrying *SIRT6* (Wang et al., 2016). Further mechanistic analysis showed that SIRT6 protected cardiomyocytes from oxidative stress in the I/R injury by activating forkhead box protein O3 (FOXO3) in an AMPK-dependent manner for upregulating FOXO-dependent antioxidant genes such as *Sod2* and *Cat*. Therefore, it is possible that this SIRT6-AMPK-FOXO3 axis may also be at work in the SIRT6 transgenic mice with NaIO_3_ described above (Yang et al., 2023).

## 5 Concluding remarks

Thus far, SIRT6 has been found to possess three different enzymatic activities: NAD^+^-dependent protein deacetylase, deacylase, and mono-ADP ribosyltransferase. With these activities, SIRT6 plays a role in a variety of biological processes for maintaining cellular homeostasis. SIRT6 functions in gene silencing as a histone deacetylase by targeting acetylated histones, such as H3K9Ac, H3K56Ac, and H3K18Ac. SIRT6 interacts with multiple biological regulators, such as NF-κB, HIF1α, PARP1, and NFE2L2 (NRF2), which dictates its dynamic localization at specific genomic sites or recruitment of specific proteins to the sites for modulating chromatin structures and thereby regulating gene expression. Beyond histones, SIRT6 also acts on non-chromatin proteins as an enzyme, and one such activity is involved in its function against oxidative stress as a coactivator of NRF2. Therefore, SIRT6 can act as both a corepressor and a coactivator in gene regulation, depending on interacting partners and biological contexts. In addition, SIRT6 contributes to genome stability and longevity by enhancing DNA repair through activation of PARP1. Furthermore, SIRT6 also plays a role in regulating SGs in the cytoplasm in response to stresses. Thus, SIRT6 has been known to play a role in many diverse biological processes, but more functions could be added to this already long list in the coming years. In most pathophysiological conditions, with some exceptions, SIRT6 exerts beneficial effects on cellular homeostasis and organismal wellbeing as described in this review. Therefore, SIRT6 has been pursued as a promising target for prevention and treatment of human diseases, such as diabetes, obesity, inflammation, neurodegeneration, and age-related diseases. To this end, substantial efforts have been made and are still ongoing to develop small molecule activators and inhibitors that can be a potential drug for SIRT6-targeted treatment in clinical settings (Huang et al., 2018; Fiorentino et al., 2021). In addition, specific modulators are useful as a tool to further elucidate molecular details of SIRT6 activity and its mechanisms through activation or inhibition of SIRT6 enzymatic activity, which holds great promise for clarifying the precise connection between SIRT6 function and observed phenotypes, as well as for further supporting SIRT6’s potential as a therapeutic target (Fiorentino et al., 2021).

In the retina and RPE, although SIRT6 was not extensively studied initially, interesting and important functions have recently emerged. Among SIRT6’s multiple functions, three are of particular interest: 1) regulation of glucose metabolism, 2) protection against oxidative stress, and 3) activation of autophagy. Retinal photoreceptor cells have unique energy (glucose) metabolism in that they rely not only on mitochondrial respiration but also aerobic glycolysis under normal conditions (“Warburg effect”) for energy production. This is because photoreceptor cells need metabolic intermediates for the daily renewal of their outer segments, a situation similar to that of continuously proliferating tumor cells. This unique metabolic need makes SIRT6 an unfavorable factor for photoreceptor cell survival, because SIRT6 represses glycolysis-related genes. Since SIRT6 generally functions as a beneficial factor for cellular wellbeing, its role in retinal photoreceptors is rather exceptional. As for its role in antioxidant defense, SIRT6 protects rat primary RGCs from H_2_O_2_-induced oxidative stress in an NRF2-dependent manner (SIRT6–BACH1–NRF2/ARE axis), as well as mouse RPE cells from NaIO_3_-induced oxidative stress *in vivo* through catalase upregulation likely in an NRF2-independent manner. Regarding the effects on autophagy, SIRT6 activates autophagy in both Aβ-stimulated cultured RPE cells and LPS-treated ARPE19 cells. Considering that oxidative stress and defects of autophagy in the RPE are among the suspected pathogenetic mechanisms of dry AMD, further studies are warranted to test whether SIRT6 could be a beneficial target for prevention, slowing progression, and/or treatment of AMD through protection against oxidative stress and activation of autophagy. Because of its myriad of functions and diverse biological processes it affects, both described and unmentioned in this review, SIRT6 is a uniquely attractive molecule that deserves further studies to better understand its roles in physiology and disease. SIRT6’s beneficial effects and therapeutic potentials have been recognized in other tissues much earlier than in the retina and RPE, and multiple small molecule activators and inhibitors have already been identified or generated as described above. This situation provides eye researchers with an advantage of learning from findings in other tissues, as well as a great opportunity to quickly test available SIRT6 modulators in retinal disease models, including culture cells, retinal organoids, and animal models. However, it is also important to exercise caution to balance SIRT6’s beneficial effects on RGCs and RPE cells with the unique metabolic need of retinal photoreceptors. Taken together, this review suggests that deeper understanding of SIRT6’s functions and their mechanisms, both inside and outside the eye, holds great promise for the future development of SIRT6-targeted therapeutic strategies for blinding eye diseases.
